# Ataxin-10 Inhibits TNF-*α*-Induced Endothelial Inflammation via Suppressing Interferon Regulatory Factor-1

**DOI:** 10.1155/2021/7042148

**Published:** 2021-11-23

**Authors:** Yong Li, Qi Zhang, Na Li, Liting Ding, Jinping Yi, Yue Xiao, Shibiao Chen, Xuan Huang

**Affiliations:** ^1^Department of Anesthesiology, The First Affiliated Hospital of Nanchang University, Nanchang, China; ^2^The National Engineering Research Center for Bioengineering Drugs and the Technologies, Institute of Translational Medicine, Nanchang University, Nanchang, China; ^3^School of Future Technology, Nanchang University, Nanchang, China; ^4^Department of Clinical Laboratory, The First Affiliated Hospital of Nanchang University, Nanchang, China; ^5^First School of Clinical Medicine, Nanchang University, Nanchang, China

## Abstract

Endothelial inflammation is a crucial event in the initiation of atherosclerosis. Here, we identify Ataxin-10 protein as a novel negative modulator of endothelial activation by suppressing IRF-1 transcription activity. The protein level of Ataxin-10 is relatively higher in human vascular endothelial cells, which can be significantly suppressed by TNF-*α* in both HUVECs and HLMECs. Overexpression of Ataxin-10 markedly inhibited the mRNA expressions of VCAM-1 and several cytokines including MCP-1, CXCL-1, CCL-5, and TNF-*α*; thus, it can also suppress monocyte adhesion to endothelial cells. Accordingly, Ataxin-10 silencing promoted endothelial inflammation. However, Ataxin-10 did not affect the MAPK/NF-*κ*B signaling pathway stimulated by TNF-*α* in HUVECs. Using the yeast two-hybrid assay, we found that Ataxin-10 can directly bind to interferon regulatory factor-1 (IRF-1). Upon TNF-*α* stimulation, Ataxin-10 promoted the cytoplasmic localization of IRF-1, which inhibited the transcription of VCAM-1. Moreover, knockdown of IRF-1 can eliminate the effect of Ataxin-10 on the expression of VCAM-1 in HUVECs induced by TNF-*α*. Taken together, these results indicate that Ataxin-10 inhibits endothelial cell activation and may serve as a promising therapeutic target for some vascular inflammatory-related diseases such as atherosclerosis.

## 1. Introduction

Atherosclerosis is a chronic inflammatory disease characterized by local accumulation of lipid-rich plaques, which is a predominant cause of cardiovascular disorders [1]. As the pathogenesis of atherosclerosis refers to multifactorial processes, increasing evidences showed that endothelial inflammation played a pivotal role in the occurrence and progression of atherosclerosis [2]. In response to inflammation, endothelial cell (EC) activation increases proinflammatory cytokine secretion and adhesion molecule expression, ultimately resulting in endothelial dysfunction and vascular inflammation [3]. Tumor necrosis factor-*α* (TNF-*α*) is a primary pathogenic mediator of EC activation [4] and also a major prototypic proinflammatory cytokine derived from activated monocytes/macrophage [5], which is mainly related to the pathogenesis of atherosclerosis [6]. TNF-*α* activates multiple cellular signaling pathways, such as the classic NF-*κ*B and MAPK pathway, to induce mRNA expression of cell adhesion molecules, including ICAM-1 (intercellular adhesion molecule 1), VCAM-1 (vascular cell adhesion molecule 1), and E-selectin, which play key roles in the crosstalk between leukocyte and endothelium [7]. Activated ECs recruit monocytes to mediate vascular inflammation, which may result in the development of atherosclerosis [8]. Besides, endothelial interferon regulatory factor 1 (IRF-1) was reported to function in the regulation of TNF-*α*-mediated signaling, especially VCAM-1 expression modulation [9]. Understanding the regulation of TNF-*α* signaling in ECs is very important to explore these therapeutic pathways.

Spinocerebellar ataxia type 10 (SCA10) is an autosomal dominant neurologic disorder which is characterized by slowly progressive gait ataxia, nystagmus, dysarthria, epilepsy, and nonmotor symptoms [10]. It is the only known human disease to date caused by ATTCT pentanucleotide repeat expansion in the *ATXN10* gene [11]. The expanded repeats cannot disrupt gene transcription [12] but bind to hnRNP K, which results in PKC *δ* translocation to the mitochondria and cell apoptosis [13]. However, *ATXN10* knockout mice are embryonic lethal, suggesting that Ataxin-10 protein is indispensable for normal embryonic development.

So far, the biological function of Ataxin-10 protein is largely unknown. Besides two armadillo repeats at C-terminus, Ataxin-10 protein does not contain other functional motifs [14]. Ataxin-10 was reported to localize predominantly in the cytoplasm and can bind to *β*2 subunit of G-protein to activate the Ras-MAP kinase-Elk-1 cascade [15]. Ataxin-10 was found to be phosphorylated by Plk1 and Aurora B in cytokinesis [16, 17]. Moreover, Ataxin-10 interacted with O-linked *β*-N-acetylglucosamine transferase (OGT) to enhance intracellular glycosylation activity, indicating that Ataxin-10 might be indispensable for intracellular O-GlcNAcylation level maintenance and homeostasis [18, 19]. Accumulating evidence shows that O-GlcNAcylation is of critical importance in mediating cardiovascular function and disease [20]. Hilgers et al. demonstrated that *O*-GlcNAcylation can inhibit TNF-*α*-induced vascular inflammation [21]. Therefore, we speculate that Ataxin-10 may regulate TNF-*α*-induced endothelial inflammation.

Here, we aimed to explore the function of Ataxin-10 protein in TNF-*α*-stimulated endothelial cell dysfunction. Using the yeast two-hybrid assay, we identified IRF-1 as a novel interactor of Ataxin-10. Furthermore, we found that Ataxin-10 exerted its anti-inflammatory effect via suppressing IRF-1 translocation to the nucleus, which resulted in reduced VCAM-1 expression and monocyte adherent. This study demonstrates a new role for Ataxin-10 in the modulation of inflammation and endothelial functions.

## 2. Materials and Methods

### 2.1. Reagents

Recombinant human TNF-*α* (94948-59-1) was bought from Sigma-Aldrich. VCAM-1 (sc-13160), ICAM-1 (sc-1511-R), Ataxin-10 (sc-271233), and *β*-actin (sc-1616) antibodies were purchased from Santa Cruz Biotechnology. I*κ*B*α* (4812), phospho-I*κ*B*α* (2859), phospho-p65 (3033), p65 (8242), phospho-IKK*α*/*β* (2078), IKK*α* (11930), IKK*β* (8943), phospho-ERK1/2 (4370), ERK1/2 (4695), phospho-JNK (4668), JNK (9252), phospho-p38 (4511), p38 (8690), phospho-C-Jun (2361), C-Jun (9165), Flag (8146), VE-cadherin (2158), IRF-1 (8478), *α*-tubulin (2125), and TBP (8515) antibodies were obtained from Cell Signaling Technology. siRNAs targeting *ATXN10* (sc-60218) were purchased from Santa Cruz Biotechnology. qPCR primers were from Integrated DNA Technologies, Inc.

### 2.2. Cell Culture and Transfection

All human primary vascular ECs including human aortic ECs (HAECs), human coronary artery ECs (HCAECs), human dermal microvascular ECs (HDMECs), human lung microvascular ECs (HLMECs), and human umbilical vein ECs (HUVECs) were purchased from Lonza Walkersville Inc. maintained in EGM™ Endothelial Cell Growth Medium BulletKit™ or EGM™-2 Endothelial Cell Growth Medium-2 BulletKit™ according to the manufacturer's instruction. For the present study, cells less than passage five were used for the experiment. THP-1 cells (human acute monocytic leukemia cell) were obtained from ATCC and cultured in 10% FBS (Sigma-Aldrich) RPMI 1640 medium (Corning), 1% penicillin/streptomycin (Invitrogen), and 0.05 mmol/L 2-mercaptoethanol (Sigma Aldrich). HEK293T cells were bought from ATCC and seeded in Dulbecco's modified Eagle's medium supplemented with 10% FBS and 1% penicillin/streptomycin (Invitrogen). Transfection of Ataxin-10 vector and siRNA into HUVECs transiently was performed by electroporation using Nucleofector device and Nucleofector kits for HUVEC (Lonza) as described [22, 23]. After 24 h, 10 ng/mL TNF-*α* was added to the cells for indicated times, and cell lysates were collected and detected by Western blotting.

### 2.3. Western Blotting and Immunoprecipitation

Mouse tissue (twelve-week-old male C57BL/6) and cells were homogenized in the lysis buffer containing a protease inhibitor cocktail (Sigma-Aldrich) as described previously [22]. Samples with equal total protein amounts (20–50 *μ*g) were separated by electrophoresis in SDS-PAGE and then transferred to the NC membrane (Corning). The membranes were blocked in 5% nonfat milk PBST (PBS with 0.1% Tween-20) for 20-30 min and then incubated with specific primary antibody overnight at 4°C with gentle shaking. After washing with PBST for three times, the blots were then incubated with secondary antibody for 2-4 h at room temperature. Bands were detected using a chemiluminescent detection kit according to the protocol (Pierce). The results were normalized to *β*-actin. The relative band density of the blots was determined with Gel-Pro analyzer software. For immunoprecipitation (IP) assays, proteins were extracted with lysis buffer and subjected to IP with Ataxin-10 or IRF-1 antibody as described previously [22].

### 2.4. qPCR

Total RNA from HUVECs was extracted using TRIzol (Invitrogen) according to the manufacturer's instructions. Then, 1 *μ*g RNA was reverse-transcribed to cDNA using the PrimeScript™ RT reagent kit with gDNA Eraser (TAKARA). Quantitative real-time PCR (qPCR) was performed in 96-well plate format using TB Green® *Premix Ex Taq*™ II (TAKARA) on ViiA-7 real-time PCR system instrument (ABI). Each reaction was performed in triplicate. As an internal control, the results were normalized to *β*-actin transcript. Fold changes were determined from cycle threshold values using the 2^-*ΔΔ*CT^ method. Sequences of the primers used here are listed in Table 1.

### 2.5. Yeast Two-Hybrid Screening

The yeast two-hybrid screening assay was performed as recommended by the manufacturer of the Matchmaker Gold Yeast Two-Hybrid System as described previously [24]. cDNA of *ATXN10* gene was cloned into the pGBKT7 vector as the bait and subsequently transformed into the yeast strain Y2HGold. The Y2HGold yeast strain was then mated with Y187 yeast strain containing a Universal Human Mate & Plate™ Library (Clontech Laboratories). Blue yeast colonies growing on SD/-Ade/-His/-Leu/-Trp/X-*α*-Gal (QDO/X/A) medium were regarded as positive candidates, and PCR was performed for the positive cDNA clones using the T7 sequencing primer followed by sequencing to identify candidate genes.

### 2.6. Isolation of Nuclear and Cytoplasm Extracts

The cytoplasm and nuclear portion were collected and separated using the Cell Fractionation Kit (Cell Signaling Technology, Inc) as previously described [25]. Briefly, cells were harvested and washed with ice-cold PBS and centrifuged at 350 g for 5 min. 500 *μ*L cytoplasmic isolation buffer was added to the pellet to fully suspend it. The suspension was kept on ice for over 5 min and then centrifuged for 5 min at 500 g. The cytoplasm in the supernatant fraction was moved to a prechilled tube and stored on ice. The pellet fraction was resuspended in 500 *μ*L membrane isolation buffer, vortexed for 15 s, then incubated on ice for 5 min, and centrifuged for 5 min at 8000 g. The pellet fraction was resuspended in 250 *μ*L cytoskeleton/nucleus isolation buffer and sonicated for 5 s. After centrifugation for 10 min at 16000 g, the supernatant including the nuclear fraction can be collected for the subsequent experiments.

### 2.7. Monocyte Adhesion Assay

The monocyte adhesion assay was carried out as previously described [22, 23]. After 24 h of transfection, cells were seeded and cultured in a 6-well plate until they reached about 85% confluence. Then, HUVECs were treated with TNF-*α* (10 ng/mL) for 8 h and washed with PBS twice. After labeling with fluorescein isothiocyanate via a PKH67 fluorescent staining kit (Zynaxis, Inc.), 5 × 10^5^ THP-1 cells were added into each well and incubated for 1 h in a 37°C incubator. Unbound cells were removed gently by washing with cold PBS. Adhesive THP-1 cells were determined using a fluorescence microscope, and the numbers were read out by the Cytation 3 Cell Imaging Multimode Reader (Biotek Instruments).

### 2.8. Statistical Analysis

Data are expressed as the means ± standard deviation (SD) of at least three independent experiments. Differences between groups were assessed by two-tailed Student's *t*-test not assuming equal variance between groups using GraphPad Prism 5.0 software. Statistical significance was defined as *p* < 0.05.

## 3. Results

### 3.1. Proinflammatory Cytokine TNF-*α* Significantly Decreases Ataxin-10 Expression in ECs

**Figure 1:**
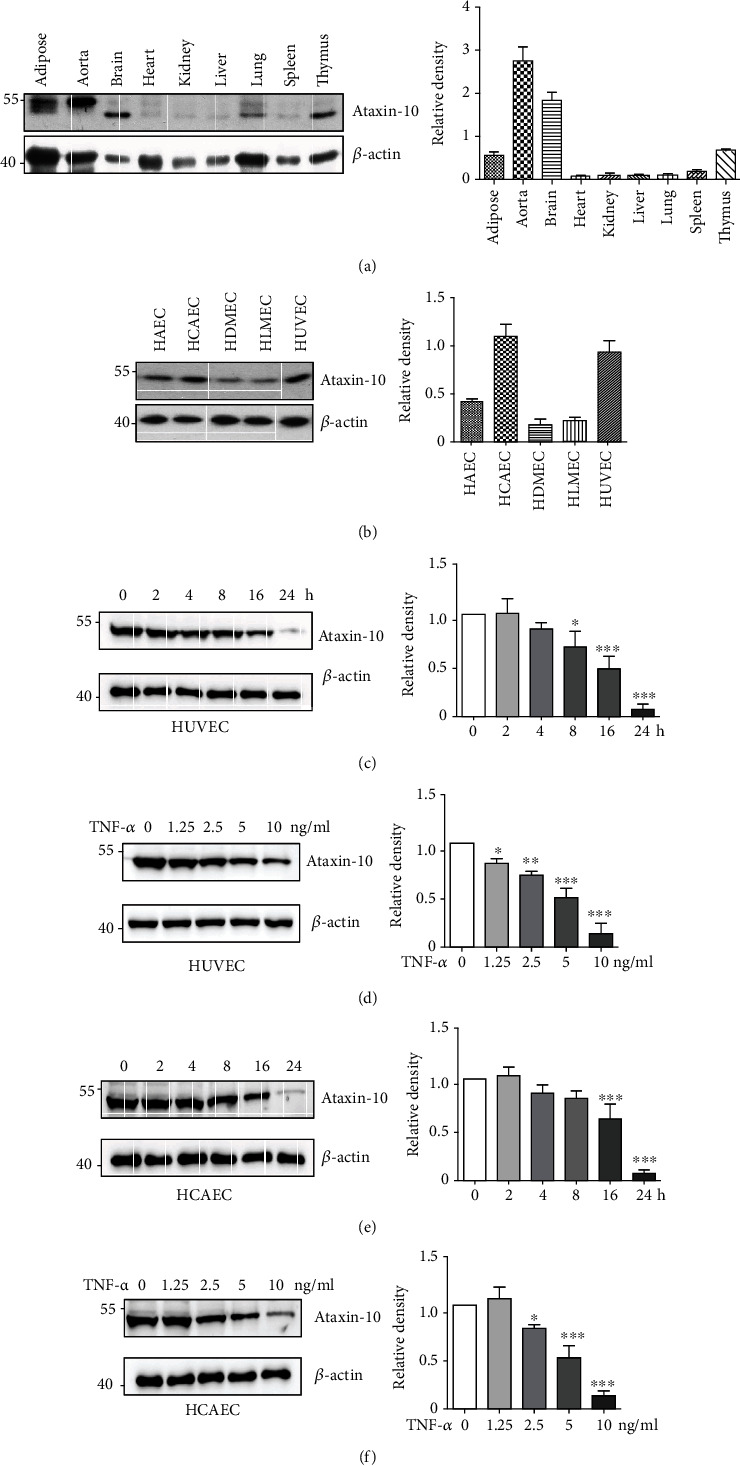
Expression of Ataxin-10 in endothelial cells. (a) Representative images of Western blotting for detection of the expression of Ataxin-10 in mouse tissues including adipose, aorta, brain, heart, kidney, liver, lung, spleen, and thymus. *β*-Actin was provided as a loading control. (b) Representative images of Western blotting for detection of the expression of Ataxin-10 in human primary vascular endothelial cells. (c, d) HUVEC or (e, f) HCAEC was stimulated with TNF-*α* for different time intervals and doses as indicated. Cell lysates were collected for Western blotting analysis. Quantification of the bands was carried out using Gel-Pro Analyzer software, and results were presented as “fold changes” at the right side of the bands.

Since the expression and function of Ataxin-10 in vascular endothelial cells were never reported, we first detected the protein expression of Ataxin-10 in a variety of mouse tissues (adipose, aorta, brain, heart, kidney, liver, lung, spleen, and thymus) and primary ECs (HAEC, HCAEC, HDMEC, HLMEC, and HUVEC). As shown in Figure 1(a), Ataxin-10 was expressed highest in the aorta among the indicated mouse tissues. Of note, the observed molecular weight (MW) of Ataxin-10 was larger in the aorta and adipose than that in the brain and thymus (Figure 1(a)), suggesting that Ataxin-10 may have posttranslational modifications in different tissues. In fact, it was reported that Ataxin-10 could be phosphorylated and O-linked glycosylated [16–18]. Ataxin-10 can be detected in all cultured primary ECs, with higher expression in HCAEC and HUVEC (Figure 1(b)). Moreover, the expression of Ataxin-10 was further inhibited by TNF-*α* in HCAECs or HUVECs in both time-dependent and dose-dependent manners (Figures 1(c)–1(f)). These results indicated that Ataxin-10 may be relevant to the regulation of vascular endothelial activation in response to TNF-*α* stimulation.

### 3.2. Ataxin-10 Inhibits Human Monocyte Adherence to TNF-*α*-Activated HUVECs

**Figure 2:**
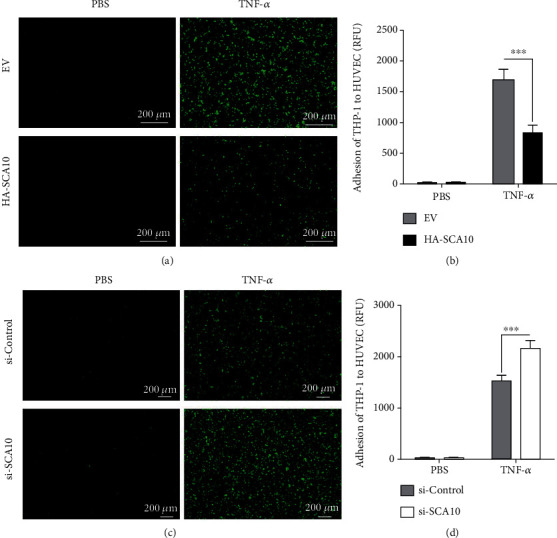
Effects of Ataxin-10 on the adhesion of THP-1 monocytes to TNF-*α*-treated HUVECs. (a, c) Photographs (×200 magnification) of PKH67 fluorescent labeled monocytes (THP-1 cells, green) adhering to HUVECs. (b, d) Quantitative data analysis revealed significant increases in adhesion of THP-1 cells to the HUVEC monolayer. The data are representative of 6 independent experiments. ^∗∗∗^*p* < 0.001 vs. TNF-*α* treated control group. (a, b) Ataxin-10 or empty vector (EV) was transfected transiently into HUVECs; 48 h later, cells were incubated with or without TNF-*α* (10 ng/mL) for 8 h. Then, fluorescence-labeled THP-1 cells were added to the activated HUVECs for 1 h. After being washed carefully by PBS, adherent cells were visualized, photographed, and counted. (c, d) The HUVECs transfected with si-control or si-Ataxin-10 were treated with TNF-*α* (10 ng/mL) for 8 h and incubated with PKH67 fluorescent labeled monocytes for 1 h. Adhesive cells were visualized, photographed, and counted as well.

Since the adhesion of inflammatory monocytes to endothelial cells is an important step in the development of atherosclerosis, we evaluated the possible role of Ataxin-10 on TNF-*α*-induced monocyte adhesion. We previously reported that TNF-*α* stimulation can markedly increase THP-1 cell adhesion to HUVECs [22, 23]. Therefore, the monocyte adhesion assay was performed after incubating with TNF-*α* for 8 h as described in Materials and Methods. As shown in Figure 2, THP-1 adhesion was minimal in unstimulated HUVECs, but treatment with TNF-*α* resulted in a marked increase of monocyte adhesion to endothelial cells. However, knocking down Ataxin-10 expression by siRNA dramatically promoted the TNF-*α*-induced adhesion between the THP-1 cells and HUVECs (Figures 2(c) and 2(d)). In contrast, overexpression of Ataxin-10 significantly inhibited the recruitment of THP-1 to activated HUVECs (Figures 2(a) and 2(b)), suggesting that Ataxin-10 has a specific inhibitory effect on monocyte adhesion induced by TNF-*α* to endothelial cells.

### 3.3. Ataxin-10 Suppresses VCAM-1 Expression in HUVECs

**Figure 3:**
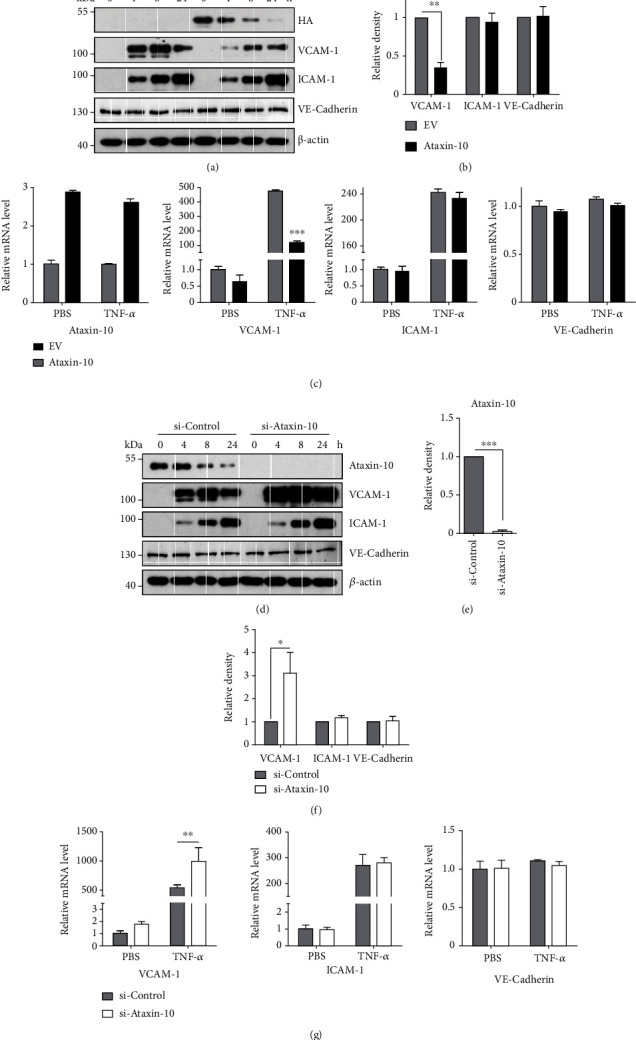
Effects of Ataxin-10 on adhesion molecule expressions in TNF-*α*-activated HUVECs. (a, d) Representative images of Western blotting for the detection of indicated protein expression. (a) HUVECs were transfected with either EV or HA-Ataxin-10 for 48 h, followed by stimulation with TNF-*α* (10 ng/mL) for the indicated times. Total cell lysates were used for immunoblotting analysis with appropriate antibodies (right panel). (b) Densitometry analysis of 3 independent experiments is shown as the relative ratio of each protein after TNF-*α* treatment (8 h) and normalized to *β*-actin. (c) HUVECs were transiently transfected with HA-Ataxin-10 or empty vector (EV) for 48 h. Then, cells were incubated with TNF-*α* (10 ng/mL) or PBS (as controls) for another 4 h. qPCR assays were performed to determine the mRNA levels of Ataxin-10 and adhesive molecules. ^∗∗∗^*p* < 0.001 vs. EV control group. (d) Ataxin-10 siRNAs or control siRNAs were transfected transiently into HUVECs. 48 h later, transfected cells were stimulated with TNF-*α* (10 ng/mL) for 0, 4, 8, and 24 h. Protein levels of VCAM-1, ICAM-1, and VE-cadherin were determined. Relative fold changes of Ataxin-10 (e) or adhesion proteins (f) after TNF-*α* stimulation (8 h) were confirmed by densitometry and normalized to *β*-actin. ^∗^*p* < 0.05 and ^∗∗∗^*p* < 0.001 vs. si-control group. (g) Ataxin-10 siRNAs/control siRNAs were transfected into endothelial cells. 24 h later, cells were incubated with TNF-*α* (10 ng/mL) or PBS (as controls) for another 4 h. qPCR assays were performed to determine the mRNA levels of adhesive molecules. ^∗∗^*p* < 0.001 vs. si-control group.

Cell adhesion is under the control of several adhesion molecules on the endothelial cell surface [8]. VCAM-1 and ICAM-1 are shown to represent predominant adhesive forces of endothelial cells under cytokine stimulation [8]. In order to explore the possible molecular mechanism of Ataxin-10 on THP-1 cell adhesion, ICAM-1 and VCAM-1 expressions were evaluated by Western blotting and qPCR. HUVECs were transiently transfected with Ataxin-10 plasmids or empty vectors for 24 h, and then, cells were treated with TNF-*α* for endothelial cell activation. The expression of adhesion molecules was detected by Western blotting and qPCR. Results of Figures 3(a) and 3(b) showed that TNF-*α* evoked a marked increase in VCAM-1 and ICAM-1 expression compared to the control group; overexpression of Ataxin-10 significantly reduced the VCAM-1 protein levels but did not affect ICAM-1 and VE-cadherin expression. Consistently, Ataxin-10 overexpression also suppressed the mRNA level of VCAM-1 but not ICAM-1 and VE-cadherin (Figure 3(c)). Next, we sought to confirm its function by determining the role of endogenous Ataxin-10 using siRNA. Confluent HUVECs were transfected with either a control siRNA duplex or a human Ataxin-10 specific siRNA. Treatment with Ataxin-10 siRNA effectively suppressed Ataxin-10 protein levels (Figures 3(d) and 3(e)). As expected, Ataxin-10 knockdown significantly promoted VCAM-1 expression in TNF-*α*-induced HUVECs (Figures 3(d), 3(f), and 3(g)). Neither ICAM-1 nor VE-cadherin were affected by Ataxin-10 knockdown (Figures 3(d), 3(f), and 3(g)). Taken together, these data indicate that Ataxin-10 specifically inhibited VCAM-1 expression induced by TNF-*α* in HUVECs.

### 3.4. Ataxin-10 Inhibits TNF-*α*-Induced Cytokine/Chemokine Production in HUVECs

**Figure 4:**
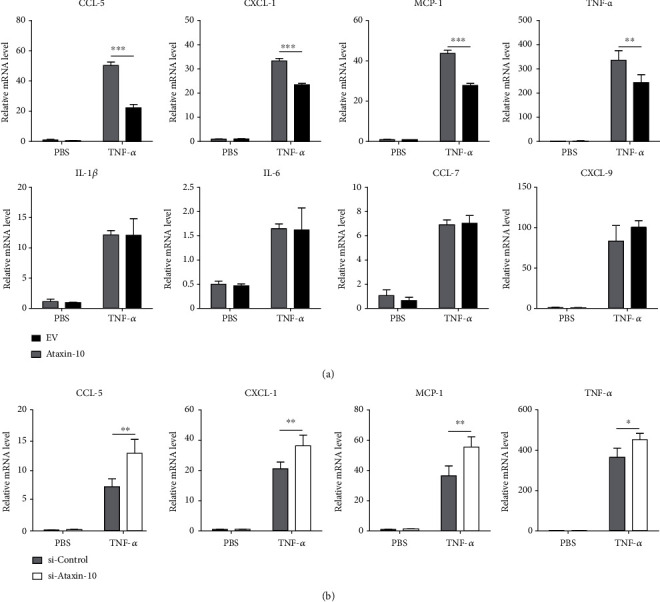
Effects of Ataxin-10 on chemokine/cytokine expression in TNF-*α*-stimulated HUVECs. (a) HUVECs were transfected with either EV or HA-Ataxin-10 for 48 h, followed by incubation with TNF-*α* (10 ng/mL) for 4 h. Relative mRNA levels of IL-1*β*, IL-6, CCL-5, CCL-7, CXCL-9, CXCL-1, MCP-1, and TNF-*α* were detected by qPCR, with *β*-actin as an internal control. ^∗∗^*p* < 0.01 and ^∗∗∗^*p* < 0.001 vs. EV. (b) Ataxin-10 was knocked down using siRNA and then stimulated with TNF-*α* for 4 h. mRNA levels of CCL-5, CXCL-1, MCP-1, and TNF-*α* were determined by qPCR. ^∗^*p* < 0.05 and ^∗∗^*p* < 0.01 vs. si-control group.

Upon TNF-*α* stimulation, endothelial cells produce proinflammatory mediators, which cause more monocytes to be recruited [26]. These proinflammatory mediators are reported to aggravate endothelial dysfunction [27]. As presented in Figure 4(a), exposure of HUVECs to TNF-*α* for 4 h significantly induced the expression of the cytokine/chemokine (including MCP-1, TNF-*α*, CXCL-1, IL-1*β*, IL-6, CCL-5, CCL-7, and CXCL-9). However, the expression of several TNF-*α*-induced chemokines (MCP-1, CXCL-1, and CCL-5) and cytokine TNF-*α* was markedly suppressed in HUVECs transfected with HA-Ataxin-10 (Figure 4(a)). IL-1*β*, IL-6, CCL-7, and CXCL-9 were not influenced by Ataxin-10 (Figure 4(a)). To further verify the regulation of these genes by Ataxin-10, a knockdown approach was applied. Ataxin-10 knockdown markedly increased the mRNA levels of MCP-1, CXCL-1, CCL-5, and TNF-*α* in HUVECs stimulated with TNF-*α* (Figure 4(b)). Again, IL-1*β*, IL-6, CCL-7, and CXCL-9 expressions were not altered (data not shown). In a word, these data identify Ataxin-10 as a negative regulator of cytokine/chemokine expression in HUVECs.

### 3.5. Ataxin-10 Does Not Affect the MAPK and NF-*κ*B Signaling Pathways Inactivating HUVECs

**Figure 5:**
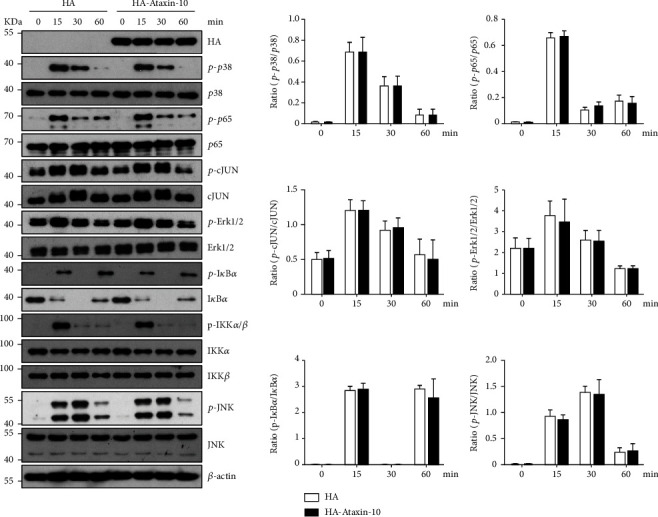
Effect of Ataxin-10 on MAPK/NF-*κ*B signaling pathway in TNF-*α*-stimulated HUVECs. HA-Ataxin-10 or empty vector was transfected into HUVECs and then stimulated with TNF-*α* for the indicated times, and whole cell lysates were prepared and harvested for immunoblotting. Blots for activated p38 (phospho-p38 Thr180/Tyr182), activated p65 (phospho-p65S536), activated c-JUN (phospho-c-JUN Ser63), activated ERK (phospho-ERK T202/Y204), activated I*κ*B*α* (phospho-I*κ*B*α*Ser32), activated IKK*α*/*β* (phospho-IKK*α*/*β*Ser176/Ser177), and activated JNK (phospho-JNK T183/Y185) are shown. Internal loading is shown by antiactin or reprobing membranes with antibodies to total p38, p65, c-JUN, ERK1/2, I*κ*B*α*, IKK*α*, IKK*β*, and JNK immunoblotting. The bands of Western blotting were quantified by Gel-Pro Analyzer software, and the results were presented as fold changes at the right side of the bands.

As we all know, MAPK/NF-*κ*B cascade plays a critical role in the pathogenesis of atherosclerosis by modulating a series of adhesion molecules and inflammation-related genes including VCAM-1 [28]. Accordingly, we examined whether Ataxin-10 affects the MAPK/NF-*κ*B signaling pathway activated by TNF-*α* in HUVECs. After transfection with HA-Ataxin-10 or empty vector (EV) for 24 h, the HUVECs were treated with TNF-*α* for different time intervals (Figure 5). As expected, TNF-*α* stimulation induced the phosphorylation of p38, c-JUN, Erk1/2, I*κ*B*α*, IKK*α*/*β*, JNK, and p65 during these experimental intervals (Figure 5). However, overexpression of Ataxin-10 did not affect TNF-*α*-induced phosphorylation of the above proteins involved in the MAPK/NF-*κ*B signaling pathway (Figure 5).

### 3.6. Ataxin-10 Inhibits IRF-1 Activity to Suppress TNF-*α*-Induced VCAM-1 in HUVECs

**Figure 6:**
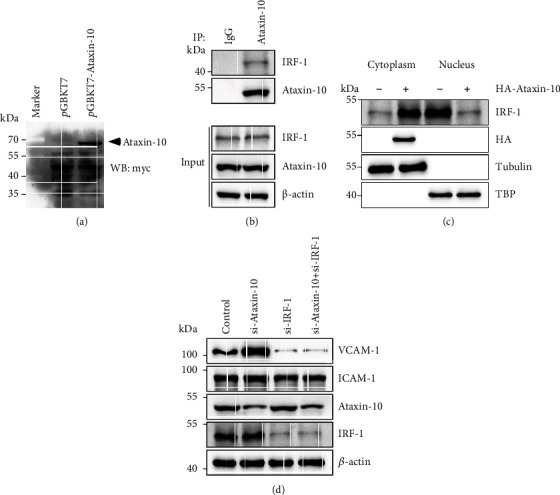
Ataxin-10 binds to and inhibits IRF-1 to suppress TNF-*α*-induced VCAM-1 in HUVECs. (a) Cell lysates were harvested from Y2HGold yeast strain transfected with pGBKT7 or pGBKT7-Ataxin-10. The expression of Ataxin-10 was detected by Western blotting. (b) HUVECs were treated with 10 ng/mL TNF-*α* for 2 hours. Immunoprecipitation experiment was performed with Ataxin-10 antibody and IgG (control), showing specific interaction between Ataxin-10 and IRF-1 in HUVECs. (c) Western blotting analysis of endogenous IRF-1 subcellular localization in HUVECs transfected with empty vector of HA-Ataxin-10. The purity and equal loading of cytoplasmic fractions and nuclear fractions were confirmed by tubulin and TBP, respectively. (d) HUVECs were transfected with Ataxin-10 siRNA or IRF-1 siRNA or both and then treated with TNF-*α* (10 ng/mL) for 8 hours. VCAM-1 and ICAM-1 protein levels were detected by Western blotting.

To gain a mechanistic understanding of the function of Ataxin-10 in TNF-*α*-induced endothelial activation, we performed yeast two-hybrid technology to screen the interaction protein of Ataxin-10. The bait vector (pGBKT7-Ataxin-10) was successfully transformed to Y2HGold (Figure 6(a)), which has no cytotoxic effect and no self-activation (data not shown). From initial putative positive colonies in the screen from the human cDNA library, one of them was identified by sequence analysis as IRF-1. To further verify the interaction between Ataxin-10 and IRF-1, a co-IP assay was performed. Since TNF-*α* stimulation of HUVECs led to a significant increase in IRF-1 protein [29], HUVECs were treated with TNF-*α* for 2 hours; then, cells were collected for protein extraction, and the cell lysates were incubated with specific anti-Ataxin-10 antibody or normal IgG. The result showed that Ataxin-10 was coimmunoprecipitated together with IRF-1 (Figure 6(b)). In addition, TNF-*α* triggered nuclear translocation and activation of IRF1 in endothelial cells [9]. As shown in Figure 6(c), quantification of protein levels by densitometry indicated that ~90% of the IRF-1 protein present in TNF-*α*-treated HUVECs localized to the nucleus. Overexpression of Ataxin-10 significantly decreased nuclear accumulation of IRF-1 (Figure 6(c)). It is reported that Ataxin-10 is mainly cytoplasmic [14]. These data suggested that Ataxin-10 may bind and sequester IRF-1 in the cytoplasm, thereby inhibiting the transcription activity of IRF-1. Using siRNA to knock down the expression of IRF-1, it can eliminate the effect of Ataxin-10 on VCAM-1 expression in HUVECs induced by TNF-*α* (Figure 6(d)). Taken together, Ataxin-10 selectively inhibits TNF-*α*-induced VCAM-1 expression in HUVECs through an IRF-1-dependent mechanism.

## 4. Discussion

Monocytes adhere to endothelial cells and then migrate to the subendothelial layer, which is a pivotal event in the early pathogenesis of atherosclerosis [30]. This is a strictly regulated process mediated by the expression of a variety of chemokines and adhesion molecules [8, 31]. Inflammatory stimuli, including TNF-*α* and other cytokines, result in endothelial cell activation and the induction of adhesive molecules [7]. Sustained adhesion and migration lead to excessive infiltration of monocytes into the arterial intima, the release of inflammatory factors, and lipid overload, which finally aggravates the instability of plaque [30]. Therefore, understanding the potential mechanism of monocyte-endothelial adherence will help to take effective strategies to prevent and treat atherosclerosis. This study proved that Ataxin-10 could reduce THP-1-HUVEC adhesion via inhibiting VCAM-1 expression. The VCAM-1 expression on the endothelial cell surface contributes to the monocyte recruitment to endothelial cells [23, 32]. Thus, Ataxin-10 may be a potential target for preventing the progression of vascular inflammatory diseases, such as atherosclerosis.

Considering the critical role of VCAM-1 in the development of atherosclerosis, considerable attention has been devoted to clarifying the regulation of VCAM-1 by cytokine in the endothelium. Ataxin-10 could inhibit TNF-*α*-induced VCAM-1 expression in HUVECs as well as its mRNA level, suggesting that Ataxin-10 may regulate VCAM-1 transcription. As MAPKs and NF-*κ*B both play significant roles in the regulation of VCAM-1 expression, we first examined whether Ataxin-10 affects VCAM-1 transcription via these signaling pathways. However, Ataxin-10 did not affect the MAPK or NF-*κ*B cascade in TNF-*α*-activated HUVECs. Through a comprehensive approach encompassing yeast two-hybrid technologies, our studies revealed that Ataxin-10 can interact with IRF-1, resulting in sequestration of IRF-1 in the cytoplasm and blocking the entry of IRF-1 into the nucleus. IRF-1 has been proved to regulate the expression of VCAM-1 [9]. It was reported that constitutively bound IRF-1 regulated by TNF-*α* is indispensable for the optimal expression of VCAM-1, which can associate with NF-*κ*B to yield a maximal response [33]. Our results indicate that regulation of Ataxin-10 in IRF-1 activity rather than MAPK/NF-*κ*B signaling pathway plays a leading role in determining the VCAM-1 expression pattern observed in response to TNF-*α* stimuli in endothelial cells.

An overexpression of inflammatory cytokines/chemokines can induce cell adhesion, migration, angiogenesis, and vascular permeability to aggravate atherosclerosis [31]. In this study, Ataxin-10 significantly reduced the mRNA expressions of MCP-1, CXCL-1, CCL-5, and TNF-*α* in TNF-*α*-stimulated HUVECs. However, the mRNA expressions of IL-1*β*, IL-6, CCL-7, and CXCL-9 were not obviously changed. It is known that the transcription of MCP-1, CXCL-1, CCL-5, and TNF-*α* was regulated by IRF-1 [34–36]. Therefore, Ataxin-10 attenuated cytokine/chemokine production in TNF-*α*-activated endothelial cells dependent on IRF-1 transcriptional activity.

## 5. Conclusions

**Figure 7:**
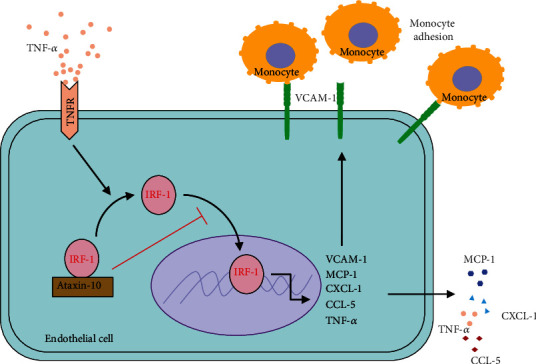
Graphic depiction of how Ataxin-10 suppresses TNF-*α*-induced VCAM-1 in endothelial cells via sequestering cytoplasmic IRF-1.

In summary, our data demonstrate a novel role for Ataxin-10 in the regulation of TNF-*α*-induced endothelial inflammation via suppressing IRF-1 (Figure 7). Our results revealed that Ataxin-10 attenuated proinflammatory changes induced by TNF-*α* in endothelial cells, which was related to downregulation of cytokines and chemokines (MCP-1, CXCL-1, CCL-5, and TNF-*α*) together with VCAM-1, resulting in a reduction in endothelial-monocyte adhesion. Interestingly, Ataxin-10 suppressed TNF-*α*-induced VCAM-1 expression but not ICAM-1/VE-cadherin expression and reduced the nuclear localization of IRF1. Our results show that the anti-inflammatory effects of Ataxin-10 are exerted on endothelial cells through suppression of IRF-1 activation.

## Figures and Tables

**Table 1:** Sequences of qPCR primers used in this study.

Primer name	Forward sequences (5′-3′)	Reverse sequences (5′-3′)
Ataxin-10	GCGGAACCGGAACTTGGATA	GCCTAAAAACTGCAGGCCAC
VCAM-1	TGTTTGCAGCTTCTCAAGCTTTT	GATGTGGTCCCCTCATTCGT
ICAM-1	AGCTTCGTGTCCTGTATGGC	TTTTCTGGCCACGTCCAGTT
VE-cadherin	ATGAGATCGTGGTGGAAGCG	TGTGTACTTGGTCTGGGTGAAG
TNF-*α*	TCTCGCACCCCGAGTGA	GGAGCTGCCCCTCAGCTT
MCP1	CAGCCAGATGCAATCAATGCC	TGGAATCCTGAACCCACTTCT
IL-1*β*	AGAAGTACCTGAGCTCGCCA	CTGGAAGGAGCACTTCATCTGT
IL-6	GGAGACTTGCCTGGTGAA	GCATTTGTGGTTGGGTCA
CXCL1	CTGGCTTAGAACAAAGGGGCT	TAAAGGTAGCCCTTGTTTCCCC
CCL-5	CGTGCCCACATCAAGGAGTA	CTTGACCTGTGGACGACTGC
CCL-7	TTGCTCAGCCAGTTGGGATTA	GCTCTCCAGCCTCTGCTTAG
CXCL-9	GAGTGCAAGGAACCCCAGTAG	AAGGGCTTGGGGCAAATTGT
*β*-Actin	ACGTTGCTATCCAGGCTGTG	GAGGGCATACCCCTCGTAGA

## Data Availability

The data used to support the findings of this study are available from the corresponding author upon request.
